# Description of the karyotype of *Sphyracephala detrahens* (Diptera, Diopsidae)

**DOI:** 10.3897/CompCytogen.v13i4.47302

**Published:** 2019-12-03

**Authors:** Ayumi Kudo

**Affiliations:** 1 Department of Biological Sciences School of Science, Tokyo Metropolitan University, Minamiohsawa 1-1, Hachiohji-shi, Tokyo 192-0397, Japan Tokyo Metropolitan University Tokyo Japan

**Keywords:** Cytogenetics, chromosomes, karyology

## Abstract

The eye stalks in Diopsidae (Bilberg, 1820) have been widely examined, but the evolutionary origin of this unique trait remains unclear. Thus, further studies of *Sphiracephala* (Say, 1828), the extant genus forming a basal branch of Diopsinae, are needed. The present study aimed to identify the karyotype of *Sphyracephala detrahens* (Walker, 1860) with conventional Giemsa staining. Cytogenetic analysis revealed a diploid number of 2n = 10 including two pairs of metacentric chromosomes, a pair of telocentric chromosomes, a pair of dot-like chromosomes, and a pair of sex chromosomes in *S. detrahens*. The congener *Sphyracephala brevicornis* (Say, 1817) has been reported to have the same diploid number, 2n = 10, but different chromosome formula. These results demonstrate that chromosome rearrangements often occur in the genus *Sphyracephala*.

## Introduction

Nearly all species of Diopsidae (Bilberg, 1820) are well-known for their exaggerated eye stalks (Shillito 1971). There are approximately 160–8000 species and 10–15 genera containing stalk-eyed flies in the family Diopsidae (Shillito 1971; Steyskal 1972; Carr et al. 2006; Ovtshinnikova and Galinskaya 2016; Roskov et al. 2019). Although both males and females in Diopsinae have eyes that are laterally displaced from the central head, the level of sexual dimorphism varies between and within species (Burkhardt and de la Motte 1985; Wilkinson and Dodson 1997; Meier and Hilger 2000). Some species of stalk-eyed flies with extreme sexual dimorphism are used as model organisms to study the evolution of sexually selected traits (Wilkinson et al. 1998; Carr et al. 2005; Husak and Swallow 2011; Knell et al. 2013). For example, in *Teleopsisdalmanni* (Wiedemann, 1830), morphology, sexual behavior, development, and cytology has been widely studied (Wilkinson and Reillo 1994; Presgraves et al. 1997; Hurley et al. 2002; Egge et al. 2011; Worthington et al. 2012; Cotton et al. 2015; Meade et al. 2019). However, limited information regarding the ecology, biology, and cytology of most stalk-eyed fly species is available, particularly for monomorphic species and primitive groups such as Sphyracepalini.

*Sphyracephala* shows the most likely ancestral state of extant Diopsinae (Kotrba 2004). *Sphyracephala detrahens* (Walker, 1860) is distributed in Taiwan, China, the Philippines, Indonesia, Papua New Guinea, and the southern islands of Japan (Ohara 1993). A few studies have examined the ecology and morphology of Japanese populations, and found the length of eye stalks less or not sexually dimorphic (Ohara 1993, 1997).

Although Baker and Wilkinson (2001) suggested that ancestral species in Diopsinae share monomorphic eye stalks, Kotrba (2004) used cladistics analysis that included the extinct species of *Prosphyracephala* to predict that sexual dimorphic eye stalks evolved in early Diopsinae. To reveal the origin of eye stalks in Diopsinae, basic studies including cytogenetic analysis of the species in Sphyracephalini need to be performed. The current study aimed to describe the karyotype of *S. detrahens* using standard chromosome staining.

## Material and methods

**Figure 1.**
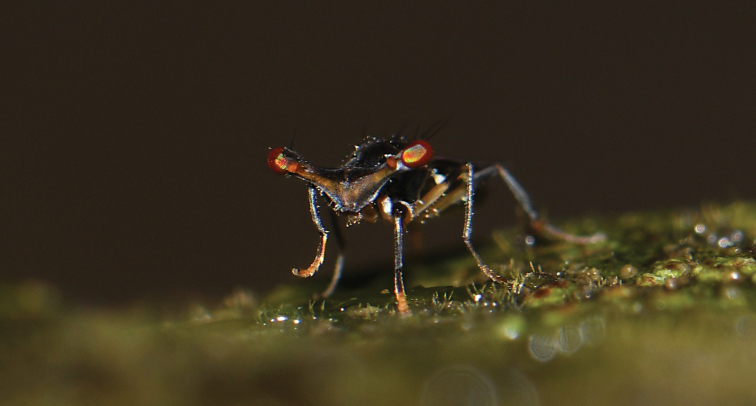
Stalk-eyed flies *Sphyracephala detrahens*.

*S. detrahens* was collected from Iriomote Island, Okinawa, Japan in April 2019 by A. Kudo (Fig. 1).All flies were maintained on organic media with yeast at 25 °C in a 14-h light:10-h dark cycle.

Metaphase chromosomes were obtained from cerebral ganglia of 3^rd^ instar larvae as described by Imai et al. (1988) without colchicine treatment. The chromosome preparations were stained with 5% Giemsa solution. The preparations were observed under a Keyence BZ-X700 fluorescence microscope (Osaka, Japan) equipped with a Nikon Plan Apo100×/1.45 oil objective and Nikon immersion Oil Type NF (Tokyo, Japan). Twenty metaphase cells with well-spread chromosomes were selected and photographed using Keyence BZ-X Analyzer software, and then processed in GIMP ver. 2. 10. 12. Fifteen individuals including 10 females and 5 males were successfully karyotyped. The length of the long and short chromosome arm was measured with Image J software ver. 1.52a (NIH, Bethesda, MD, USA). These data were used to calculate the chromosome index and arm ratio, following which chromosome classification and idiogram construction were performed as described by Levan et al. (1964).

**Table 1.** Morphometric parameters of *Sphyracephala detrahens* chromosomes from mitotic metaphase plates.

**Chromosome**	**Length of short arm (mean ± SE µm)**	**Length of long arm (mean ± SE µm)**	**Total length of Chromosome (mean ± SE µm)**	**Arm ratio^†^**	**Centromeric index^‡^**	**Chromosome classification** ^§^
**1**	4.11 ± 0.15	4.45 ± 0.15	8.56 ± 0.29	1.08	48.0	m
**2**	2.49 ± 0.09	2.94 ± 0.10	5.43 ± 0.18	1.18	45.9	m
**3**	–	–	3.95 ± 0.14	–	–	t
**4**	–	–	0.66 ± 0.02	–	–	d
**X**	1.63 ± 0.06	3.56 ± 0.11	5.19 ± 0.17	2.18	31.5	sm
**Y**	1.80 ± 0.18	2.13 ± 0.24	3.93 ± 0.41	1.18	45.9	m

^†^ Arm ratio = length of long arm/length of short arm; ^‡^ Centromeric index = length of short arm/total length of chromosome; ^§^ Chromosome classification; m: metacentric chromosome; sm: submetacentric chromosome; t: telocentric chromosome; d: dot-like chromosome.

## Results and discussion

**Figure 2.**
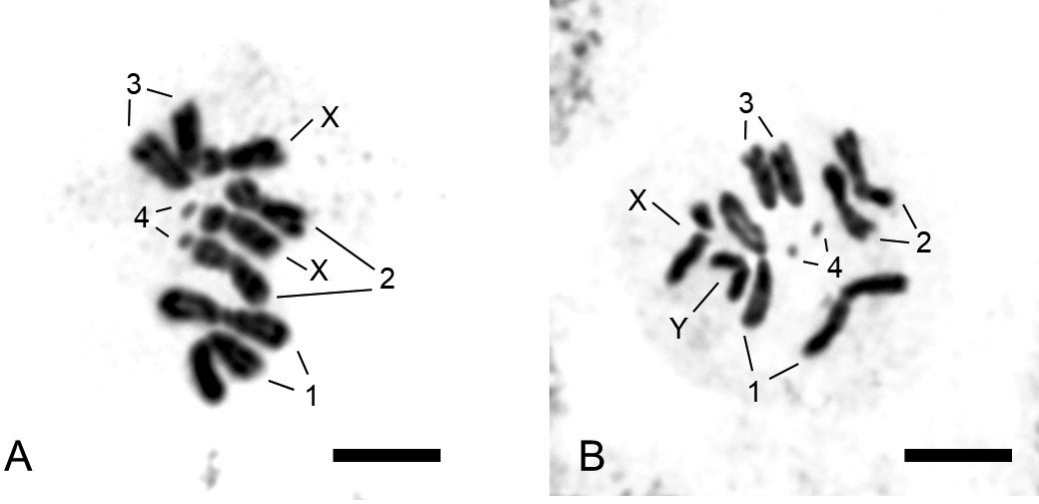
Mitotic metaphase of *Sphyracephala detrahens* with 2n = 10 chromosomes **A** female **B** male. Scale bars: 5µm.

**Figure 3.**
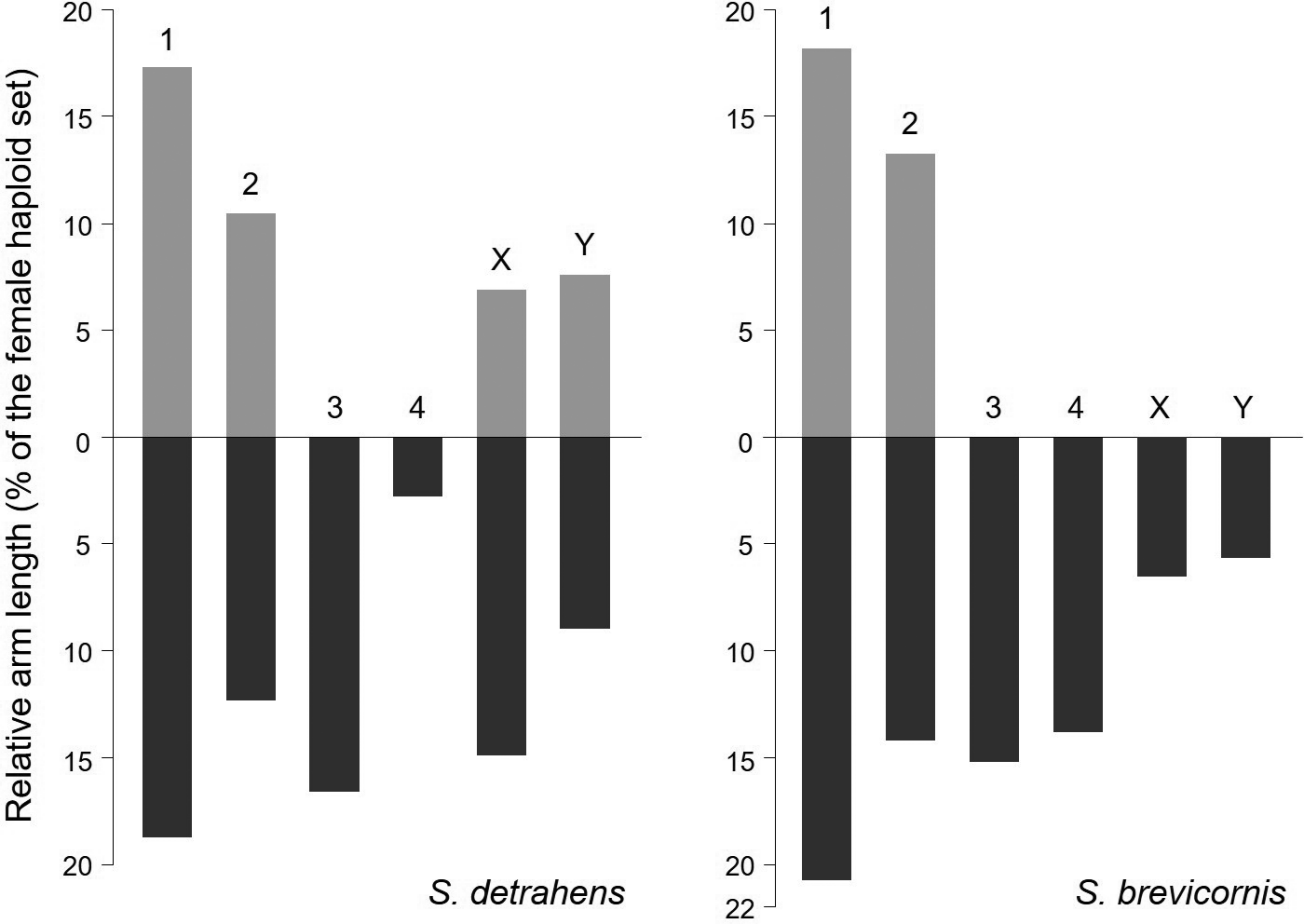
Idiograms of *Sphyracephala detrahens* and *Sphyracephala brevicornis*. The numbers above each bar indicate chromosome numbers. The light and dark regions represent short arms and long arms, respectively. Idiograms of *S. brevicornis* were modified and redrawn from Idiogram 1 of *S. brevicornis* (Jan 1966).

This is the first study to reveal that the chromosome number of *S. detrahens* was 2n = 10 (Fig. 2). The karyotype of *S. detrahens* consisted of two pairs of metacentric chromosomes, a pair of rod-shaped telocentric chromosomes, a pair of dot-like microchromosomes, and a pair of sex chromosomes (Figs 2, 3). In the female cerebral ganglia cells, a homomorphic sex chromosome pair was formed by the two submetacentric X-chromosomes (Fig. 2A). In the male cerebral ganglia cells, a heteromorphic pair of sex chromosomes was formed by the X-chromosome and metacentric Y-chromosome (Fig. 2B). The Y-chromosome was slightly stained and was shorter than the X-chromosome (Fig. 2).

Although a congener, *S. brevicornis*, had the same diploid chromosome number 2n = 10, the karyograms of *S. brevicornis* differed from that of *S. detrahens* (Fig. 3); the karyotype of *S. brevicornis* consisted of two pairs of metacentric chromosomes, two pairs of telocentric chromosomes, and a pair of small telocentric XY pair (Jan 1966). The sex chromosomes showed the greatest differences between the two species. Both the X and Y chromosomes in *S. detrahens* were bi-armed and larger compared to those in *S. brevicornis*. Thus, chromosomal rearrangements occurred in these two species and their relatives. Information about the phylogenetic relationships between *S. detrahens* and its congeners has been never analyzed. Further investigations into phylogenetic relationships will aid in the understanding of differences in karyograms between *S. detrahens* and *S. brevicornis*. Despite the lack of karyological information in Diopsinae, comparative cytogenetic analyses using related species will lead to a greater understanding of chromosomal evolution in stalk-eyed flies.
